# Treatment of Anaemia in Patients with Acute Burn Injury: A Study of Blood Transfusion Practices ^†^

**DOI:** 10.3390/jcm10030476

**Published:** 2021-01-27

**Authors:** Ioana Tichil, Samara Rosenblum, Eldho Paul, Heather Cleland

**Affiliations:** 1 Department of Oncology and Haematology, “Iuliu Hatieganu” University of Medicine and Pharmacy, 73 21 December Boulevard, 400603 Cluj-Napoca, Romania; 2 Department of Anatomy and Embryology, “Iuliu Hatieganu” University of Medicine and Pharmacy, 3-5 Clinicilor Street, 400006 Cluj-Napoca, Romania; 3 Victorian Adult Burns Service, Alfred Hospital 55 Commercial Road, Melbourne, VIC 3004, Australia; S.Rosenblum@alfred.org.au (S.R.); H.Cleland@alfred.org.au (H.C.); 4 Department of Epidemiology, Preventive Medicine School of Public Health, Preventive Medicine Monash University, 553 St Kilda Road, Melbourne, VIC 3004, Australia; eldho.paul@monash.edu; 5 Central Clinical School, Monash University, The Alfred Centre, 99 Commercial Road, Melbourne, VIC 3004, Australia

**Keywords:** blood transfusion, burn surgery, anaemia in burns, transfusion triggers, risk factors, outcomes

## Abstract

*Objective*: To determine blood transfusion practices, risk factors, and outcomes associated with the use of blood products in the setting of the acute management of burn patients at the Victorian Adult Burn Service. *Background*: Patients with burn injuries have variable transfusion requirements, based on a multitude of factors. We reviewed all acute admissions to the Victorian Adult Burns Service (VABS) between 2011 and 2017: 1636 patients in total, of whom 948 had surgery and were the focus of our analysis. *Method and results*: Patient demographics, surgical management, transfusion details, and outcome parameters were collected and analyzed. A total of 175 patients out of the 948 who had surgery also had a blood transfusion, while 52% of transfusions occurred in the perioperative period. The median trigger haemoglobin in perioperative was 80mg/dL (IQR = 76–84.9 mg/dL), and in the non-perioperative setting was 77 mg/dL (IQR = 71.61–80.84 mg/dL). Age, gender, % total body surface area (TBSA) burn, number of surgeries, and intensive care unit and hospital length of stay were associated with transfusion. *Conclusions*: The use of blood transfusions is an essential component of the surgical management of major burns. As observed in our study, half of these transfusions are related to surgical procedures and may be influenced by the employment of blood conserving strategies. Furthermore, transfusion trigger levels in stable patients may be amenable to review and reduction. Risk adjusted analysis can support the implementation of blood transfusion as a useful quality indicator in burn care.

## 1. Introduction

The etiology of post-burn anaemia is multifactorial, including blood loss due to destruction at the time of injury, low grade chronic bleeding from unhealed wounds, surgery, and multiple blood tests. Intrinsic causes include sequestration, haemolysis, nutritional deficiencies, and dysfunctional erythropoiesis [1]. Coagulopathies due to various causes are also common after burns, and are associated with increased transfusion requirements [2]. These anaemias and coagulopathies, especially in critically ill patients, frequently require transfusion of blood products, and patients with severe burns may have massive transfusion requirements. Despite this, the specific effects, for example, on wound healing and infective complications, of anaemia and transfusions in burns patients, and optimal transfusion protocols, are currently unknown.

Studies of transfusion practices in general populations of critically ill patients, which usually exclude burns patients, have indicated no worse, and possibly improved, outcomes when restrictive protocols are followed; the CRIT study (a prospective, multi-center, observational cohort study of intensive care unit patients) demonstrated decreased length of intensive care unit (ICU) and hospital stay, mortality, and complication rates in patients who received fewer blood transfusions [3]. More liberal transfusion protocols are considered however in the case of patients suffering from chronic conditions, such as coronary artery disease or chronic kidney disease, due to the negative impact of anaemia [4,5].

Studies in burns patients have been mostly of a retrospective nature; however, protocols emphasizing the need for blood conservation during surgery, and involving the use of defined “trigger points” for transfusion, are associated with decreased blood use [6,7,8].

The only prospective randomized trial examining the relationship between transfusion and outcomes to date in burns patients was conducted by Palmieri et al. [9]. This was a multi-center trial involving patients with >20% total body surface area (TBSA) burns who were allocated to a restrictive (transfusion at Hb <7 g/dL) or a liberal (transfusion at <10 g/dL) transfusion group. The restrictive group received fewer transfusions, but there was no associated decrease in infective complications, organ failure, or mortality, and outcomes were not different between the two groups.

In addition to consideration of the effects of different transfusion protocols on clinical outcomes and blood product use, transfusion in burns patients may also be considered an indicator of quality of care, particularly as it relates to surgical management. Blood use is related to number of surgeries, timing of surgery, operative time, and infectious complications, in a complex interaction between injury, patient, and treatment factors. Patients with extensive burns who have early meticulous surgery focusing on removal of non-viable tissue, blood preservation, and staged strategic wound closure employing biosynthetic skin substitutes for wound closure have better outcomes, and fewer blood transfusions [6,10,11,12], findings from earlier studies notwithstanding [13].

The aims of this study were to determine blood transfusion practices, risk factors, and outcomes associated with the acute management of burn patients at the Victorian Adult Burn Service (VABS), in order to better understand factors associated with transfusions, and identify opportunities for better blood stewardship in our population. Considering recently published data advocating restrictive transfusion protocols, we hypothesize that current transfusion levels in practice are higher than optimal, and therefore this review was also intended to inform consideration on the value of benchmarking of blood product use in adult burn patients in order to aid in formulating guidelines for the surgical management of burns patients.

## 2. Methods

This retrospective study was registered with The Alfred Ethics Committee, project no 509/17, and was considered for low risk review, and approved on 16 October 2017. It included patients admitted to VABS with acute burn injury during a 6-year period between July 2011 and July 2017, and who underwent excisional surgery. Exclusion criteria were patients admitted more than 28 days post-injury (primarily patients presenting for secondary non-acute procedures); patients with multi-trauma and minor burns (<5%TBSA); patients deemed to have non-survivable injuries, who were not actively treated; and those with recorded pre-existing anaemia of chronic illness.

Eligible patients were identified from prospectively collected data using the VABS database, and the Burns Registry of Australia and New Zealand (BRANZ). These registries provided demographic data, including patient age and sex, mechanism of burn, date of injury, %TBSA burn, intensive care unit (ICU) length of stay, surgical management, and ventilation requirements. The presence and severity of inhalation injury was also extracted from the VABS database, and was diagnosed using a standardized bronchoscopy scoring system based on the abbreviated injury score, as previously reported [14].

Patients with associated cardiac comorbidities such as ischemic heart disease, congestive cardiac disease, cardiomyopathy, and hypertensive disease were identified using ICD-10 comorbidity codes and electronic medical records. Coagulopathy was defined as international normalized ratio (INR) >1.5 or activated partial thromboplastin time (APTT) >60 s [15]. All laboratory results were electronically extracted from hospital databases.

All transfused blood products strictly adhered to Australian National Blood Authority Patient Blood Management, Australian and New Zealand Society of Blood Transfusion, and Australian Red Cross Guidelines. All fresh blood products underwent mandatory ABO and RhD blood type testing, viral and red cell antibody screening, as well as syphilis testing. Leucodepletion was performed for all red cell and platelet products. Bacterial contamination screening was conducted for platelets stored at room temperature. Red cells were only stored at 2–6 °C in temperature-controlled dedicated blood refrigerators for a maximum of 42 days. Platelets were stored at 20–24 °C for up to 5 days. Fresh frozen plasma, cryodepleted plasma, and cryoprecipitate were stored at or below –25 °C for up to 12 months.

Red blood cell (RBC) use for each patient was obtained from the hospital blood bank database. All haemoglobin (Hb) laboratory values determined throughout each patient’s admission were analyzed. For each patient, a mean value of all these was determined, and recorded as average Hb. Minimum and maximum Hb levels throughout admission were also collected for all patients, as well as Hb levels on admission and discharge. In order to identify triggers and targets for transfusion, “transfusion events” were analyzed. A transfusion event was defined as the sequential transfusion of red blood cells extending over a maximum period of 72 h, with less than 12 h between each RBC unit. Transfusion events were either perioperative or non-perioperative. Perioperative transfusions were defined as occurring within 24 h prior to surgery and up to 48 h post-surgery; thus, covering a perioperative interval of 72 h. Transfusions outside this interval were classified as non-perioperative.

The “trigger” for transfusion was defined as the Hb immediately prior to the unit being administered, and the “target” was defined as the first Hb level post transfusion “event”. We acknowledge that while Hb levels are often essential to the decision to transfuse a patient, many other factors are considered, such as clinical setting, patient status, physician experience, as well as national and international guidelines. For the purpose of this study we chose to look at Hb levels as objective and universally accepted triggers for blood transfusions.

Outcome data included blood culture results, occurrence of deep venous thrombosis, organ dysfunction, mortality, and length of stay. Organ dysfunction included cardiac, renal, and liver dysfunction. Cardiac dysfunction was defined as a rise in troponin levels over 0.4 ng/mL [16]. Renal dysfunction was defined as kidney injury according to RIFLE (risk, injury, failure, loss) criteria and AKI (acute kidney injury) KDIGO (kidney disease improving outcomes) stage, above [17,18]. Hepatic dysfunction was defined as a bilirubin level greater than 2 mg/dL [19,20].

Data were analyzed using SAS software version 9.4 (SAS Institute, Cary, NC, USA). Continuous variables were summarized using mean (standard deviation) or median (interquartile range), depending on the distribution of the data. Categorical variables were expressed as counts and proportions. Comparison between groups (transfused versus not transfused) were made using the Student’s t-test for normally distributed continuous variables, Wilcoxon rank-sum test for non-normally distributed continuous variables, and chi-square or Fisher’s exact test, as appropriate, for categorical variables. Multivariate analysis for transfusion was performed using multiple logistic regression, whereas hospital length of stay was analyzed via multiple linear regression, with results reported as odds ratios (95% confidence intervals) or regression coefficients (standard errors), where appropriate. As hospital length of stay had a positively skewed distribution logarithmic, transformation was applied prior to multivariate analysis. Variables with a *p* value <0.05 on univariate analysis or those judged to be clinically significant were considered for inclusion in multivariate models. All calculated *p* values were two-tailed, and *p* < 0.05 indicated statistical significance.

## 3. Results

Between July 2011 and July 2017, there were 1636 acute burns admissions. Of these 1636 admitted patients, 948 underwent excisional surgery for treatment of their burn. Of these, 175 patients had a blood transfusion. Subsequent analysis was conducted on patients having excisional surgery. Characteristics of these patients are detailed in Table 1.

### 3.1. Transfusion Data

**Figure 1:**
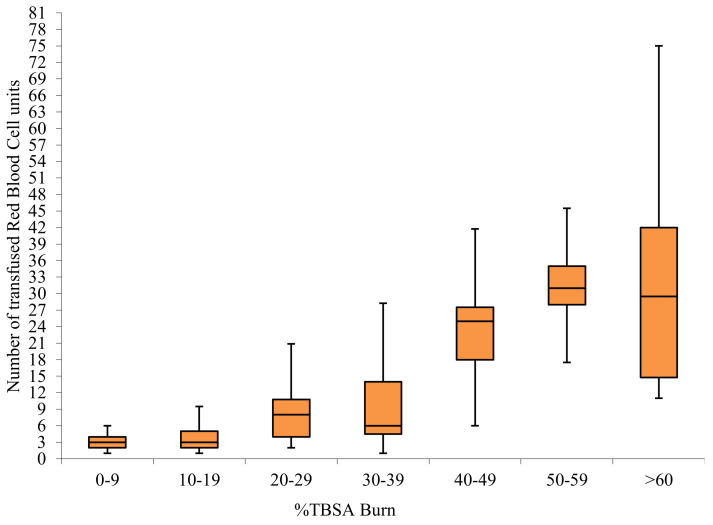
Red blood cell usage by TBSA in transfused surgical patients.

A total of 1890 RBC units, 341 plasma units, 69 platelet units, and 3 units of cryoprecipitate were transfused in the surgical group throughout the period of the study. The average number of RBC units given to transfused surgical patients by size of burn is shown in Figure 1.

Analysis of transfusion data showed differences in Hb determinations throughout hospital stay between the transfused and non-transfused groups (Table 1). In the transfused population, average haemoglobin (Hb) on admission was 147 mg/dL (IQR = 124–162), and 100 mg/dL (IQR = 92–108) on discharge. While, 52% of transfusions occurred within the perioperative timeframe.

**Figure 2:**
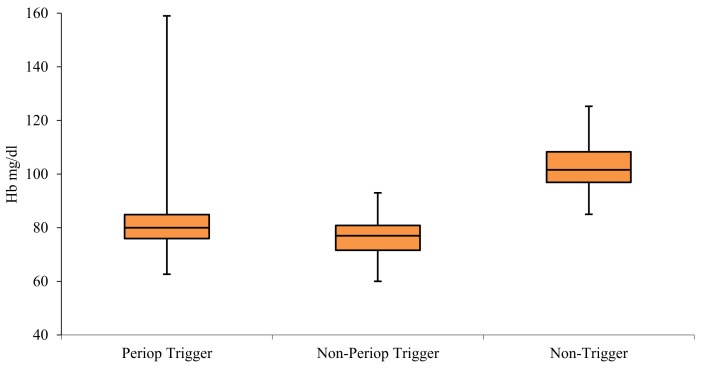
Comparison of perioperative and non-perioperative haemoglobin triggers for transfusion, and average of lowest haemoglobin values that did not trigger a transfusion in surgical patients.

Perioperative trigger Hb levels for transfusion were more variable than those for non-perioperative transfusions (Figure 2). Anaemia associated with surgical blood loss triggered blood transfusions when mean Hb levels reached 80 (IQR = 76–84.9) mg/dL. The mean non-perioperative threshold for transfusions was slightly lower at 77 (IQR = 71.61–80.84) mg/dL. Hb levels above 100 mg/dL uncommonly triggered transfusions. Transfusion targets within the perioperative and non-perioperative time frame were similar at 93.67 (IQR 88.63–98.38) mg/dL and 91.9 (IQR 87–97.75) mg/dL, respectively.

**Figure 3:**
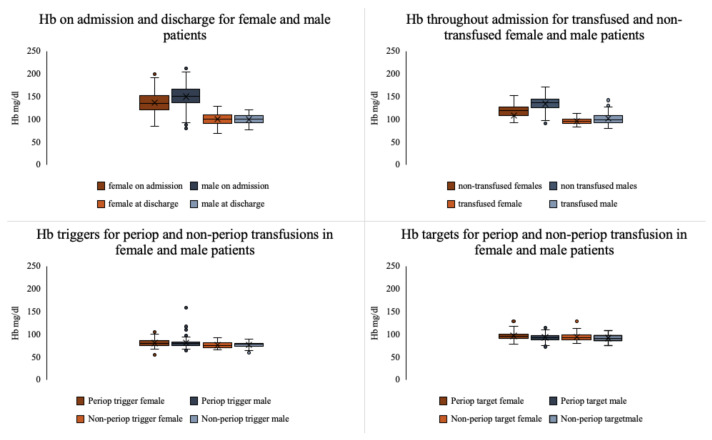
Comparison of haemoglobin values throughout admission, and transfusion triggers and targets for female and male patients.

Differences between transfused and non-transfused patients by gender are outlined in Table 2. Female patients received throughout admission a mean of 5 (IQR = 3–14) RBC units, and men were transfused with a mean of 4 (IQR = 2–13) RBC units. Moreover, 23% of female patients with burn injuries who had surgery also required a blood transfusion, whereas a total of 17% of surgical male patients were transfused. Hb triggers and targets for transfusion by gender and Hb levels throughout admission are outlined in Figure 3.

### 3.2. Risk Factors for Transfusion

Results of multiple logistic regression analysis (Table 3) showed that age, gender, % TBSA burn, number of surgeries, associated coagulopathy, and the length of ICU stay were independently associated with transfusion in this study.

### 3.3. Outcome Analysis

Multivariate analysis of length of hospital stay showed that transfusion was independently associated with increased length of stay (regression coefficient 0.511, standard error 0.085; *p* < 0.001) after adjusting for age, %TBSA burn, and comorbidities. A similar multivariate analysis of organ dysfunctions failed to show an association with transfusions (OR = 1.06, 95% CI: 0.62–1.83; *p* = 0.83)

## 4. Discussion

The Australian National Blood Authority Patient Blood Management Guidelines emphasize that the decision to transfuse should not be based on Hb alone, but on patient status. In stable patients, practice should be to transfuse a single unit and reassess. Many of the guidelines are described essentially as “practice points” only, because, beyond an evidence-based recommendation that a restrictive strategy should be employed in the critically ill, it was considered that there was insufficient evidence regarding precise trigger points and targets for transfusions.

Our study shows somewhat restrictive Hb triggers outside of theatre episodes 7.7 g/dL (IQR 7.16–8.08), and more liberal thresholds in perioperative setting of 8 g/dL (IQR 7.6–8.5) (Figure 2). Patients with burns who required surgical treatments included in this study were a heterogeneous group of varying degrees of complexity, ranging from young healthy individuals who tolerate injury and surgery well and recover uneventfully, to those with co-morbidities who become critically ill, require multiple surgeries, and have a prolonged course to recovery or death. Although the average triggers are within the acceptable ranges consistent with practice points of the National Blood Authority, and risk factors for transfusion (for example, age) have clinical validity, there is considerable variation in practice with respect to both target and trigger Hb levels. This variation may be appropriately addressed with specific guidelines for burns patients, and it is likely that this will result in decreased blood use [6,9].

Several papers have provided data on the average number of blood units transfused per patient by size of burn [10,21,22,23,24]. Graves et al. reported an average of 19.7 RBC units transfused per patient with burns >10%TBSA; Vasko et al. 8.94 units <30%TBSA, and a minimum of 17 units transfused for burns >30%TBSA; Palmieri et al. reported 13.7±1.1 RBC units in burns >20%TBSA, and a minimum of 30 units for burns >50% TBSA; Yogore et al. reported usage of 12 ± 3 RBC units per patient with burns above 20%TBSA, and higher than 20 units in burns >40%TBSA. Our data (Figure 1) shows similar usage: an average of 9.6 units used in >20%TBSA burns, and 29.4 units in >40%TBSA. However due to differing reporting methods, comparison is not precise, and for blood use to function as a quality indicator, data need to be further risk adjusted based on %full thickness burn, age, cardiac co-morbidities, and hematological conditions. More than half of transfusions occurred in the perioperative time frame, and can be expected to be minimized by application of surgical techniques associated with decreased blood loss. This provides an opportunity to benchmark quality of care as reflected in blood transfusion, through the Burns Registry of Australia and New Zealand (BRANZ), which is a clinical quality registry to which all Australian and New Zealand specialist burns units contribute [25].

In our study, age, gender, %TBSA burn, number of surgeries, coagulopathy, and ICU length of stay were independently associated with blood transfusions, in keeping with previous reports [2,26]. Inhalation injury and cardiac comorbidity have been previously linked to increased risk of transfusion [27], however, our statistical model was unable to demonstrate this association in our patient population. Male gender was associated with a decreased risk of transfusion, and this persisted when cause of burn was included in the analysis. This finding appears not to have been reported previously. Transfused and non-transfused females included in our study had comparable characteristics in terms of demographics, injury, surgical management, and time to recovery to the male population, as outlined in Table 2. Throughout admission women generally displayed lower baseline Hb levels compared to the male population (Figure 3), however Hb triggers and targets for transfusion were equivalent, potentially leading to the increased usage of blood products in the treatment of women. Wu et al. showed increased plasma transfusion in burned females [26], and a paediatric study reported increased blood loss during surgery in males [11]. “Overtransfusion” of women has previously been identified in association with elective surgical procedures [28], and we have reported increased mortality in female critically ill burns patients [29]. There are gender differences in aspects of burn care, including transfusion requirements, however, at present these differences are insufficiently well understood and require further research.

Transfusions, age, gender, %TBSA burn, number of surgeries, and the presence of cardiac comorbidities were associated with increased length of hospital stay; and age, gender, %TBSA burn, and comorbidities, but not transfusion, were associated with organ dysfunction. There were too few fatalities or bacteraemias to analyze. While observational studies have supported the idea that there is a relationship in the burns patient between transfusion and infection risk [10], the only randomized trial thus far conducted has not shown this to be the case. Palmieri et al. [21] reported increased risk of mortality and infection in transfused patients in a multicenter retrospective cohort analysis, however their prospective multicenter randomized trial [9] did not show shorter length of stay, improved rates of mortality, organ dysfunction, nor infection in patients who received fewer transfusions.

In addition to the retrospective nature of our study we believe other limitations may be represented by not being able to accurately include data on burn depth, time to first surgery, and body mass index for all patients, as well as information on storage duration for all transfused blood products. Such data may provide further insights into risk factor analysis.

## 5. Conclusions

Blood products remain essential to the management of anaemia in burns patients. Our paper identifies several risk factors leading to higher transfusion requirements, and we consider that transfusion thresholds may be amenable to reduction, specifically in the female population in order to minimize associated risks and complications. As such, blood use in burns should be an ongoing area of focus, as well as a quality of care indicator.

## Figures and Tables

**Table 1:** Summary of all surgical patient characteristics.

Variable	Transfused (175)	Non-Transfused (773)	*p* Value
Age (years)	52.6 ± 19.3	43.3 ± 18.7	<0.001
Gender (Male)	66.9% (117)	75% (580)	0.027
TBSA (%)	20 (12, 35)	5 (2, 10)	<0.001
Cause Scald	12% (21)	27.9% (216)	<0.001
Cause Chemical	0.6% (1)	5.4% (42)	0.005
Cause Contact	3.4% (6)	8.4% (65)	0.024
Cause Flame	80.6 (141)	52.3% (404)	<0.001
Cardiac Comorbidity	43.4% (76)	12.3% (95)	<0.001
Coagulopathy	41.7% (73)	2.7% (21)	<0.001
Inhalation injury	22.3% (39)	3.2% (25)	<0.001
ICU stay (hours)	165.6 (0, 405.6)	0 (0, 0)	<0.001
Ventilation (hours)	60 (0, 232)	0 (0, 0)	<0.001
Time to first surgery (days)	0.95 (0.41, 4)	3.09 (1.15, 6.9)	<0.001
Number of surgeries	3 (2, 5)	1 (1, 1)	<0.001
Time to last surgery (days)	21,06 (8.64, 40.92)	4.75 (1.81, 8.9)	<0.001
Length of stay (days)	34.28 (23.28, 62.1)	10.1 (5.99, 14.81)	<0.001
Mortality	6.9% (12)	0.4% (3)	<0.001
Positive blood cultures	17.1% (30)	0.8% (6)	<0.001
Deep venous thrombosis	6.9% (12)	0.5% (4)	<0.001
Renal dysfunction	25.7% (45)	3.8% (29)	<0.001
Cardiac dysfunction	45.7% (80)	16.6% (128)	<0.001
Hepatic dysfunction	14.3% (25)	1.6% (12)	<0.001
Minimum Hb (mg/dL)	69.6 ± 11.9	119.3 ± 20.1	<0.001
Average Hb (mg/dL)	99.9 ± 10.8	131.1 ± 15.3	<0.001
Maximum Hb (mg/dL)	157.4 ± 28.6	145.9 ± 15.6	<0.001

Data expressed as mean ± standard deviation, medians (25th, 75th percentiles) or percentage (number). TBSA = total body surface area. ICU = intensive care unit.

**Table 2:** Summary of transfused and non-transfused patients by gender.

Variable	Transfused Females (58)	Transfused Males (117)	Non-Transfused Females (193)	Non-Transfused Males (580)
Age (years)	55 ± 20	51 ± 19	44.4 ± 18.6	42.9 ± 18.7
TBSA (%)	19.5 (10, 32)	20 (12, 37)	4 (1.5, 8)	5.5 (2, 11)
Cardiac comorbidity	46.5% (27)	41.8% (49)	16.5% (32)	11% (64)
Coagulopathy	41.3% (24)	41% (48)	3.60% (7)	2.40% (14)
Number of surgeries	3 (2, 4)	3 (2, 5)	1 (1, 1)	1 (1, 1)
Average Hb (mg/dL)	96 ± 6.6	101.8 ± 11.9	120.4 ± 12.8	134.4± 14.4
Minimum Hb (mg/dL)	67.5 ± 9.2	70.6 ± 12.9	110.3 ± 16.2	122 ± 20.4
Length of stay (days)	33.6 (27.1, 51.9)	35.4 (22, 62.8)	9.5 (4.75, 13.9)	10.3 (6.27, 14.8)
ICU stay (hours)	175.2 (2.4, 352.8)	148.8 (0, 477.6)	0 (0, 0)	0 (0, 0)

Data expressed as mean ± standard deviation, medians (25th, 75th percentiles) or percentage (number).

**Table 3:** Transfusion risk factors.

Variable	Odds Ratio	Lower 95% Confidence Limit	Upper 95% Confidence Limit	*p* Value
Age	1.03	1.02	1.05	<0.0001
Gender (Male)	0.48	0.27	0.87	0.016
TBSA	1.09	1.06	1.13	<0.0001
Number of surgeries	4.18	2.91	6.01	<0.0001
Inhalation	0.75	0.28	1.99	0.57
Coagulopathy	4.55	1.96	10.53	0.0004
Cardiac comorbidity	1.39	0.74	2.60	0.30
ICU hours	1.01	1.00	1.01	<0.0001

## Data Availability

The data presented in this study are available on request from the corresponding author. The data are not publicly available due to privacy reasons.
